# Autophagy drives epidermal deterioration in a Drosophila model of tissue aging

**DOI:** 10.18632/aging.100549

**Published:** 2013-04-10

**Authors:** Christoph Scherfer, Violet C. Han, Yan Wang, Aimee E. Anderson, Michael J. Galko

**Affiliations:** ^1^ Department of Biochemistry and Molecular Biology, The University of Texas MD Anderson Cancer Center, Unit 1000, Houston, TX 77030, USA; ^2^ Genes & Development Graduate Program, The University of Texas MD Anderson Cancer Center, Unit 1000, Houston, TX 77030, USA

**Keywords:** epidermis, autophagy, Drosophila, healthspan

## Abstract

Organismal lifespan has been the primary readout in aging research. However, how longevity genes control tissue-specific aging remains an open question. To examine the crosstalk between longevity programs and specific tissues during aging, biomarkers of organ-specific aging are urgently needed. Since the earliest signs of aging occur in the skin, we sought to examine skin aging in a genetically tractable model. Here we introduce a *Drosophila* model of skin aging. The epidermis undergoes a dramatic morphological deterioration with age that includes membrane and nuclear loss. These changes were decelerated in a long-lived mutant and accelerated in a short-lived mutant. An increase in autophagy markers correlated with epidermal aging. Finally, the epidermis of *Atg7* mutants retained younger characteristics, suggesting that autophagy is a critical driver of epidermal aging. This is surprising given that autophagy is generally viewed as protective during aging. Since *Atg7* mutants are short-lived, the deceleration of epidermal aging in this mutant suggests that in the epidermis healthspan can be uncoupled from longevity. Because the aging readout we introduce here has an early onset and is easily visualized, genetic dissection using our model should identify other novel mechanisms by which lifespan genes feed into tissue-specific aging.

## INTRODUCTION

Life expectancy is currently increasing in both developed and developing countries. In turn, age-related health problems represent a growing socioeconomic challenge for society. To date, the interplay of life expectancy and healthy aging of different organs is not well understood. In the past, genetic model systems like the fruit fly *Drosophila* have allowed the identification of evolutionarily conserved genes that control longevity [1, 2], such as insulin-like peptides [3] and TOR signaling [4, 5]. These studies have generally used lifespan of population cohorts as a primary readout. However, we still know little about the control of organ aging or organ “healthspan” in individual aging animals. Healthspan is defined as the period of time in an organism's life during which its physiological good health is maintained, and is currently viewed by many as a more important readout of aging than lifespan [6]. Importantly, many tissues in a multicellular organism age differently at the cellular level. For example, *C. elegans* muscles show early signs of deterioration, while the nervous system remains remarkably intact [7]. This likely reflects the fact that individual tissues make different contributions to lifespan, suggesting that their healthspans may be regulated differently. Hence, an open question about the relationship between lifespan and healthspan is how longevity genes differentially control individual tissue-specific aging programs. To examine this issue, simple and early visual tissue biomarkers of “aging in progress” are urgently needed. Recently, physiological biomarkers of aging have been identified in the *Drosophila* heart [8], muscles [9], nervous system [10] and sleep patterns [11]. However, most of these organs and behaviors either have a complex morphology and are not easily visualized from the exterior, or are not easily measured and only show changes that arise after a week or two into adulthood. A more easily accessible and earlier tissue aging readout would thus provide a valuable model for further studies on organ aging.

The skin shows the first obvious changes with age of any organ. Altered skin morphology is externally visible and is influenced by both extrinsic environmental factors as well as the intrinsic aging program. In humans, some of these changes include a gradual atrophy and thinning of the epidermal layer by up to 50% from the age of 30 to 80 years [12]. In addition to externally visible changes, both membrane protein levels [13] and global gene expression profiles [14] are known to change in aging skin. Because the skin sits at the interface between the organism and its environment it is a promising tissue to search for biomarkers of aging. A genetically tractable *Drosophila* model of skin aging would provide numerous advantages, including short lifespan, a simple monolayer epidermis, and a powerful genetic toolbox for manipulation and visualization.

Recently, research has suggested that one of the most important cellular processes to impact aging is autophagy, the process by which an intracellular membrane engulfs organelles and cytoplasmic material to form an autophagosome which is then digested after fusion with a lysosome [15]. Autophagy maintains tissue homeostasis by clearance of aggregates of damaged proteins and other molecules. This function is particularly important in the nervous system [16], where the intricate cell morphology renders the cells especially sensitive to the accumulation of aggregates. Autophagy can also lead to cell death [15] and is important for cellular remodeling processes [17-19]. In the context of aging, autophagy is often seen as protective, since it reduces damaged organelles that accumulate with age [20]. Moreover, senescent keratinocytes in culture die by autophagy, and blocking autophagy delays this process [21]. Whether autophagy can also drive tissue remodeling or deterioration in aging skin remains unclear.

Here we introduce the adult epidermis of the fly as an early onset tissue readout for the aging program. Age-related changes in this model include a loss of epidermal membrane labeling followed by a decrease in the number of nuclei. We also observed a strong thinning of the epidermal layer with age. Importantly, our study reveals that epidermal aging is a plastic process that is decelerated in a long-lived fly mutant and accelerated in a Progeria-like mutant with shortened lifespan. Deceleration of epidermal aging in an autophagy mutant suggests that autophagy is a driving force behind age-related alterations in the fly epidermis, in contrast to its protective role in other tissues.

## RESULTS

### Age-related morphology changes in the adult epidermis

**Figure 1:**
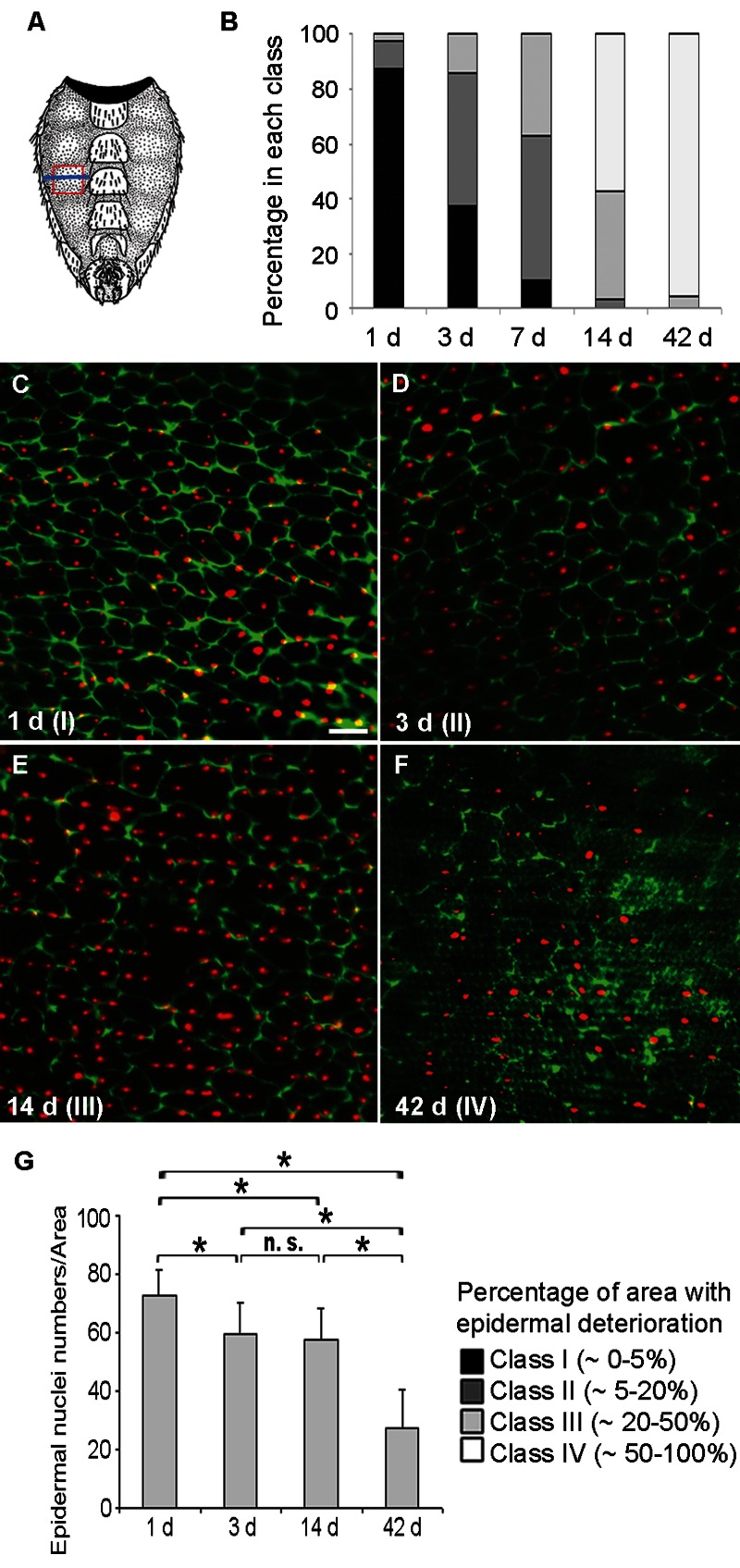
Loss of Membranes and Nuclei in the Aging Adult Epidermis (**A**) Schematic of the ventral adult abdominal epidermis (reprinted with kind permission by Cold Spring Harbor Laboratory Press from [56]). Red frame, section of the pleura analyzed by immunofluorescence; Blue line, approximate location of TEM cross-sections. (**B**) Quantification of epidermal deterioration in control w1118 flies with age (n ≥ 10). See **C-F** for a representative example of each morphology class. (**C-F**) Control (*w;UAS-DsRed2-Nuc2/CyO; NP2108-GAL4/TM6C*) epidermal whole mounts of different ages expressing nuclear DsRed2-Nuc (red) and labeled with anti-Fasciclin III (green). Bar, 20 μm. C, 1 d. D, 3 d. E, 14 d. F, 42 d. Green channel intensity elevated in F for visualization of weakly-labeled membranes. All comparisons between different time points were significantly different using the Chi square test (p < 0.05). (**G**) Quantification of nuclear numbers in epidermal whole mounts of flies of different ages bearing *NP2108-GAL4* and *UAS-DsRed2-Nuc* (n = 7 for 1 d and 14 d; n = 8 for 3 d and n = 3 for 42 d). Asterisks, significant comparisons by Single-Factor Anova (p < 0.05); n. s., not significant.

We first tested if *Drosophila* adult epidermal morphology changes with age. The membranes of the ventro-lateral abdominal epidermis (see Figure 1A) were visualized with a Fasciclin (Fas) III antibody (Figure 1C-F). In 1d old flies the epidermis was a uniform monolayer of mononuclear cells (Figure 1C). Surprisingly, although laboratory-reared flies can live for several months, loss of epidermal membrane labeling was apparent within a few days of eclosion (Figure 1D) and progressed steadily so that very little epidermal membrane labeling remained by six weeks (Figure 1F). A comparable deterioration was also observed using a FasIII-GFP fusion construct or anti-Coracle labeling (data not shown). To quantify the morphological changes in control flies we defined four classes reflecting an increasing loss of epidermal membrane labeling (see Figure 1C-F). All pair wise comparisons of class distributions at 1, 3, 7, 14, and 42 days were significant (Figure 1B). Co-labeling of epidermal nuclei revealed that these initially persisted despite the loss of epidermal membranes (Figure 1D, E). In contrast, by six weeks of age, epidermal nuclei were also strongly decreased in number (Figure 1F and G).

**Figure 2:**
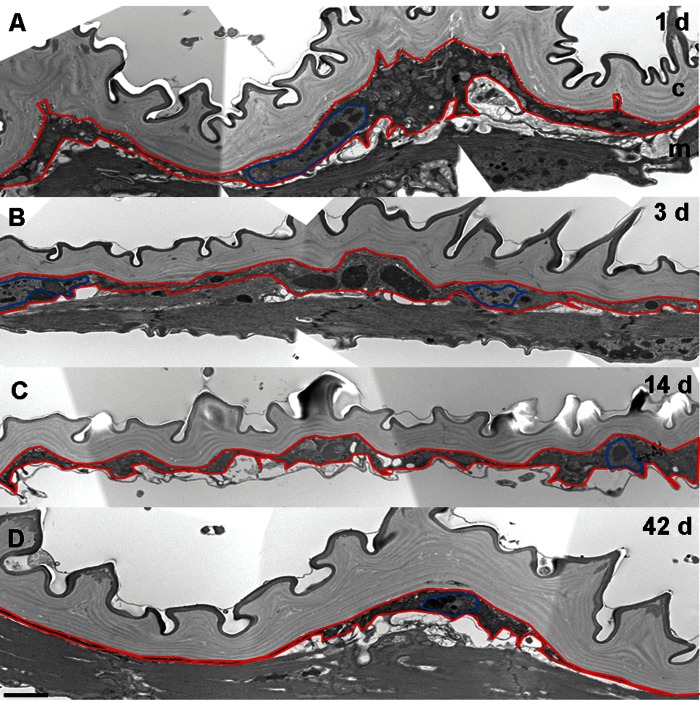
Epidermal Thickness Decreases in Aging Flies (**A-D**) TEM analysis of ventral pleura in w1118 controls. Bar, 2 μm. A, 1 d. B, 3 d. C, 14 d. D, 42 d. Red, epidermal boundaries; Blue, epidermal nuclei. c, cuticle; m, muscles. The presence of muscles underlying the epidermis varies with the precise plane of section and was not dependent on age.

We next examined epidermal changes at the structural level by transmission electron microscopy (TEM) in control flies (Figure 2). On day one, a continuous epidermal monolayer (average thickness 1.34 μm, n = 4) (Figure 2A) was observed distinct from the underlying muscles. A strong loss of cytoplasmic volume was detected as early as 3 d (average thickness 1.03 μm, n = 5), followed by a further gradual reduction at later time points (Figure 2B-D). From 3 to 14 days epidermal nuclei were condensed, while at 42 days clearly defined nuclei were often absent. Despite these other markers of epidermal deterioration, a thin but continuous epidermal layer and its basal lamina were maintained even at 42 d. Together, these fluorescence and TEM data suggest that progressive changes in the epidermis occur as the fly ages, and may represent one of the earliest reporters of insect tissue aging.

### A gene that prolongs lifespan decelerates epidermal aging

We next asked if epidermal aging would be altered in a mutant affecting *Drosophila* lifespan. Decreasing insulin signaling prolongs lifespan in diverse organisms [3, 22-26].

**Figure 3:**
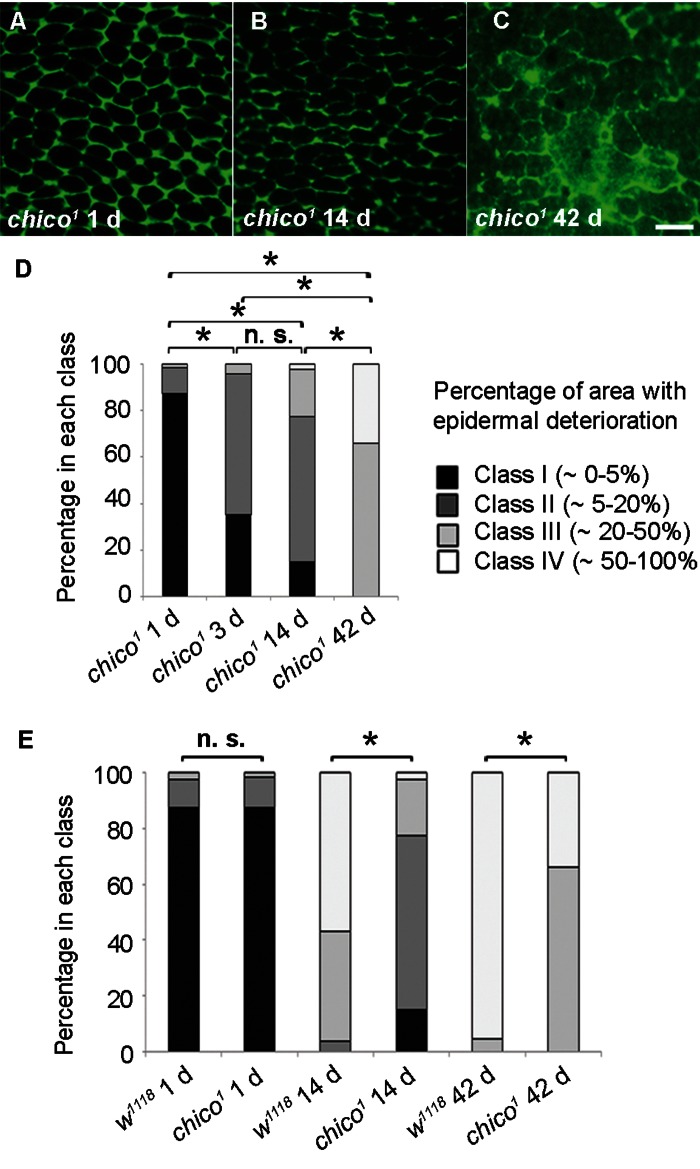
Decelerated Epidermal Aging in Long-lived chico Mutants (**A-C**) Anti-Fasciclin III immunofluorescence (green) of chico1 epidermal whole mounts (n ≥ 10). Bar, 20 μm. A, 1 d. B, 14 d. C, 42 d. (**D**) Quantification of chico1 epidermal deterioration with age. Asterisks, significant comparisons by Chi square test (p < 0.05); n. s., not significant. (**E**) Quantitative comparison of epidermal deterioration in chico1 mutants and w1118 controls. Significance indicators as in Figure 3D.

In *Drosophila*, flies that are homozygous mutant for the *insulin-like receptor substrate* (*chico*) have increased lifespan [27-29]. Therefore, we assessed markers of epidermal aging in *chico^1^* flies. On day one, epidermal morphology in *chico^1^* mutants was indistinguishable from controls (Figure 3A, E). However, epidermal membrane labeling over time was strongly preserved (Figure 3A-C) in comparison to controls (compare Figure 3B and C to Figure 1E and F). Morphological class distributions at 14 and 42 days were significantly different from age-matched controls (Figure 3E). Indeed, the 14 d old *chico^1^* mutant epidermis most closely resembled 3 d old controls and the 42 d old *chico^1^* mutant epidermis still looked younger than that of 14 d old controls. By TEM, the decrease in cytoplasmic volume and epidermal thickness also appeared to be decelerated (Figure S1A-C). These results are consistent with the idea that *chico^1^* mutants, which have an extended lifespan [27] also retain the characteristics of a younger epidermis.

### A mutant with shortened lifespan exhibits accelerated epidermal aging

**Figure 4:**
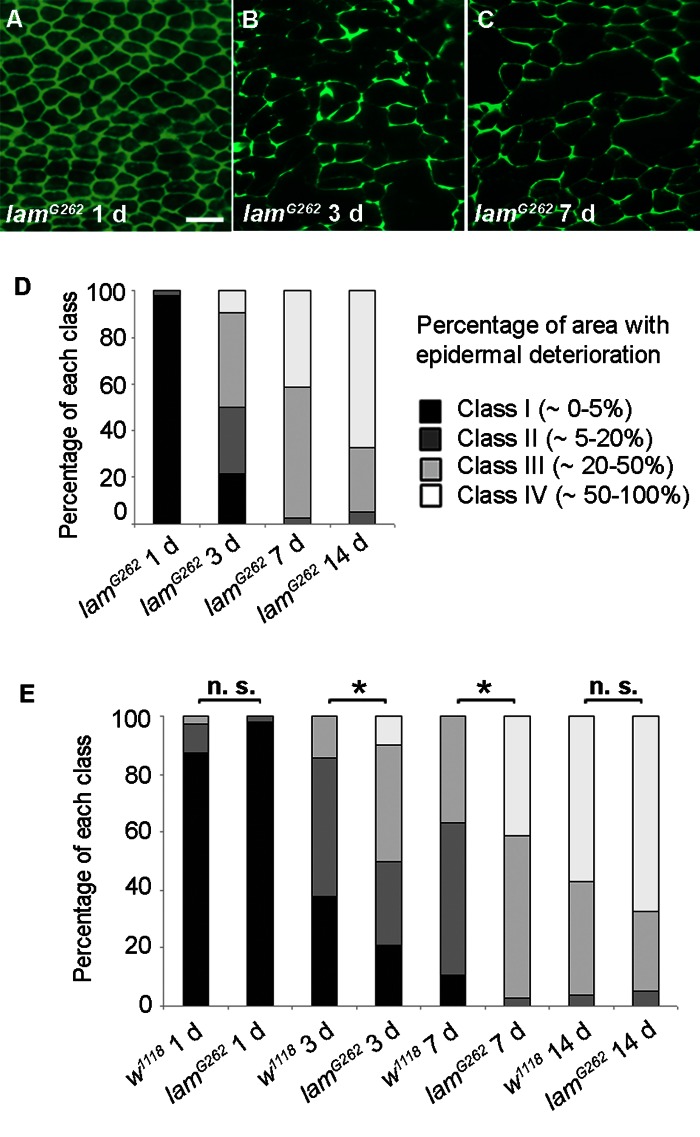
Epidermal Aging is Accelerated in Short-lived lamin Mutants (**A-C**) Anti-Fasciclin III immunofluorescence (green) of *lam^G262^* epidermal whole mounts (n ≥ 10). Bar, 20 μm. A, 1 d. B, 3 d. C, 7 d. (**D**) Quantitative comparison of epidermal deterioration in *lam^G262^* mutants and w1118 controls. All comparisons between different time points were siginificantly different using the Chi square test (p < 0.05). **E**, Quantitative comparison of epidermal deterioration in *lam^G262^* mutants and w1118 controls. Significance indicators as in Figure 3D.

Conversely, we investigated if a mutation that shortens lifespan would affect epidermal aging. In humans, disorders that resemble premature aging, including atypical Werner syndrome and Hutchinson-Gilford progeria syndrome, have been linked to mutations in genes encoding lamin proteins [30-32]. The premature aging phenotype in these disorders is accompanied by a reduced lifespan. Whether laminopathy disorders truly represent accelerated aging is vigorously debated [33] but it is clear that many of these diseases are characterized by an early onset of gene expression changes and morphological skin changes resembling those of normal aging [34]. We thus investigated if a mutation in a *Drosophila* lamin gene would alter epidermal morphology. Although many lamin mutant alleles do not survive to the adult stage, adult escapers of a B-type lamin allele, *lam^G262^*[35], have a shortened lifespan [36]. In 1 d old *lam^G262^* flies epidermal morphology resembled controls (Figure 4A), suggesting that development of the adult epidermis during pupariation proceeded normally. By 3 days and 7 days, the *lam^G262^* mutant epidermis showed more morphological deterioration than control epidermis of the same age (Figure 4B and C) and the classification of *lam^G262^* mutant epidermal pictures (Figure 4D) supported an accelerated epidermal deterioration at these time points compared to controls (Figure 4E). In 14 d old *lam^G262^* flies epidermal morphology did not look significantly different. Finally, TEM revealed strong nuclear condensation at 3 days compared to control nuclei at this time point (Figure S2B). Although there was no obvious difference in epidermal thickness in 3 d old *lam^G262^* mutants compared to age-matched controls, TEM samples of *lam^G262^* mutants exhibited altered cellular morphology. In summary, these results suggest that epidermal aging is accelerated at early time points in *lam^G262^* mutants compared to control flies.

### Autophagy correlates with and drives epidermal deterioration

**Figure 5:**
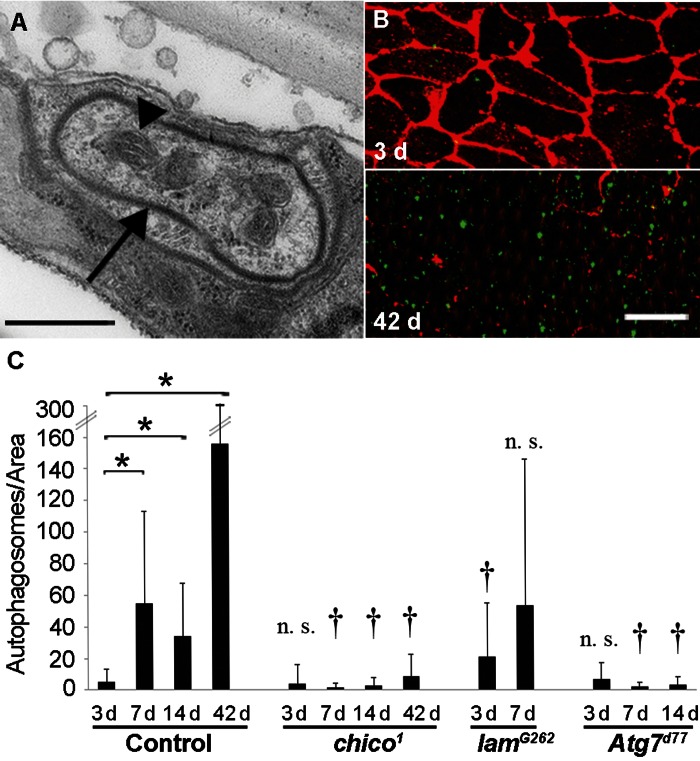
Autophagy Levels Correlate with Epidermal Deterioration (**A**) TEM of autophagosome (arrow) in 14 d old w1118 epidermis. Bar, 500 nm. Arrow, autophagosome membrane. Arrowhead, Mitochondrion. (**B**) 3 d (top) and 42 d (bottom) old control (*NP2108-GAL4* and *UAS-LC3-GFP*) epidermal whole mounts labeled with anti-Fasciclin III (red) and LC3-GFP signals amplified with anti-GFP (green). Bar, 20 μm. (**C**) Quantification of autophagosome numbers (using *NP2108-GAL4* and *UAS-LC3-GFP*) in epidermal whole mounts of the indicated genetic backgrounds. (n ≥ 20 for each time point). Asterisks, statistically significant comparisons (Single-factor Anova test (p < 0.05)) of control time points; Daggers, statistically significant comparisons of mutants versus controls of the corresponding time points; n. s., not significant.

The dramatic loss of membrane and cytoplasm in aging epidermal cells suggested that autophagy might drive the observed changes in morphology. We thus examined the aging epidermis for markers of autophagy. Consistent with the hypothesis that autophagy was responsible for the loss of membrane and cytoplasm, we found large autophagosomes in 14 d old control flies (Figure 5A). We next quantified epidermal autophagosomes at different ages and in different genotypes using a transgene, *UAS-LC3-GFP*, that labels autophagosomal membranes. We compared 3 d and older flies to avoid high autofluorescence levels in the 1 d old epidermis. Autophagosome numbers increased significantly from 3 d to 42 d in controls (Figure 5B and C). As expected, autophagosome numbers remained low in *Atg7^d77^* flies with disrupted autophagy [37] and in *chico^1^* mutants regardless of age. By contrast, autophagic activity on day 3 (but not day 7) was higher in *lam^G262^* flies than in controls. In conclusion, an early rise in autophagy levels (lamin mutant) compared to controls correlates with accelerated epidermal aging. Conversely, a decrease in autophagy levels (*chico* or *Atg7* mutants) correlates with decelerated epidermal aging.

**Figure 6:**
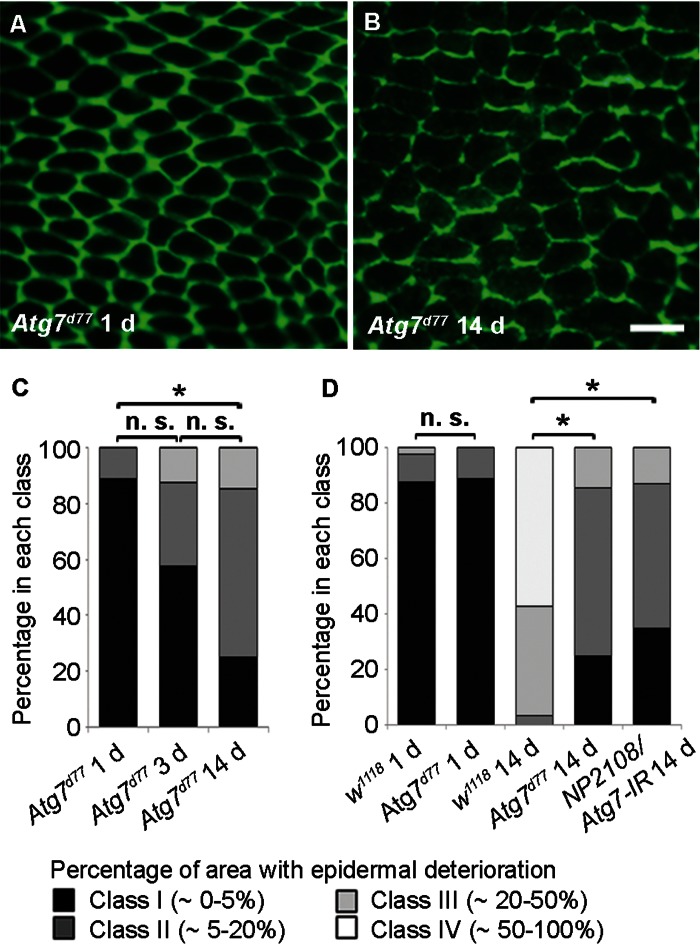
Autophagy is Required for Epidermal Aging (**A-B**) Anti-Fasciclin III immunofluorescence (green) of *Atg^7d77^* epidermal whole mounts (n ≥ 10). Bar, 20 μm. A, 1 d. B, 14 d. (**C**) Quantification of *Atg^7d77^* epidermal deterioration with age. Asterisks, significant comparisons by Chi square test (p < 0.05). n. s., not significant. (**D**) Quantitative comparison of epidermal deterioration in *Atg^7d77^* mutants, *NP2108-GAL4/Atg7-IR* flies and w1118 controls. Significance indicators as in **C**.

These findings prompted us to test if autophagy drives age-related epidermal morphology changes since autophagy can drive developmental [38] and physiological remodeling of tissues [39]. Like *chico^1^* and *lam^G262^* mutants, the initial epidermal morphology of *Atg7^d77^* flies resembled controls (Figure 6A). Interestingly, at 14 d loss of epidermal membrane labeling was strongly decelerated in comparison to controls (compare Figure 6B to Figure 1E and Figure 6C to Figure 1B). Additionally, TEM analysis revealed a thicker epidermal layer in 14 d old *Atg7^d77^* flies compared to control epidermis of the same age (Figure S4).

To test if Atg7 controls epidermal aging in a tissue-autonomous manner, we used *NP2108-GAL4* to express an RNAi targeting *Atg*7. *NP2108-GAL4* is expressed in the adult epidermis. It is also expressed transiently in the larval fat body at eclosion, but this expression persists less than 24 hours and is therefore unlikely to affect epidermal aging at later stages (Figure S5). No expression in the adult fat body was observed. Epidermal knockdown of Atg7 resulted in decelerated epidermal aging similar to the mutant, suggesting a tissue-autonomous role for autophagy (Fig. 6D). Together, these results suggest that changes in epidermal morphology with age are driven by autophagy.

## DISCUSSION

Here we establish an adult *Drosophila* model of epidermal aging in a search for early healthspan biomarkers and the genes that regulate them. By 3 d post-eclosion, earlier than other established biomarkers of tissue aging, both loss of epidermal membrane staining and cytoplasmic volume were apparent. Later, some epidermal nuclei were lost as well. Because blockade of insulin signaling decelerated epidermal aging and a *Drosophila* lamin mutant with shortened lifespan accelerated epidermal aging, age-related changes in this tissue are analogously regulated by these two established aging pathways. Surprisingly, we find that in contrast to its protective role in many aging models autophagy is driving morphological deterioration of the *Drosophila* adult epidermis (comparisons of epidermal morphology quantifications from different time points and genotypes are compiled in Figure S3). The autophagy mutant that exhibited decelerated epidermal aging is itself short-lived [40], suggesting that in this tissue healthspan can be uncoupled from lifespan, at least in the case of the cellular process that drives tissue aging. We discuss this and other curious features of our model in more detail below.

### Complex Roles for Autophagy in Tissue Aging

In the context of aging of the organism as a whole, it is generally believed that autophagy is primarily protective [20]. Increased autophagy correlates with increased lifespan in worms [41] and normal levels of systemic autophagy are required for the lifespan extension observed upon reduction of insulin signaling [42]. In *Drosophila*, maintenance of a sufficiently high level of autophagy in the nervous system and muscles seems to be particularly important for lifespan extension since neuronal-specific induction of autophagy leads to healthier neurons and lifespan extension [16, 43]. The role of autophagy in other aging tissues such as the epidermis has not been investigated until now. Unexpectedly, we observe that autophagy both correlates with and drives morphological deterioration of an aging adult tissue. Our results highlight the complex relationship between autophagy and the aging of individual tissues. Given the contrasting roles of autophagy in neuronal and epidermal tissue aging we hypothesize that the relationship between tissue-aging and lifespan may be unique for each organ. In other words, the net effect of autophagy on lifespan is likely determined by its particular role in those tissues most critical for survival. Specifically, we propose that in tissues whose healthspan is essential for setting lifespan, such as the nervous system, autophagy is protective, while some other tissues whose healthspan is not critical, such as the epidermis, may be degraded by autophagy.

While it is expected that all organs will deteriorate with age, it is surprising that this seems to be an actively regulated process in the case of the adult fly epidermis. Why? One possibility is that once the cuticle barrier structure is formed shortly after eclosion the adult epidermis becomes dispensable for survival. Our TEM data suggests that cuticle secretion stops after two to three days into adulthood (data not shown) and the expression of known adult cuticle proteins ceases around this same time [44]. The majority of epidermal degradation we observed occurred after cuticle secretion was complete, suggesting that aging, rather than development, drives the degradation process. We hypothesize that the adult fly normally degrades its epidermis as a nutrition source and that autophagy orchestrates this process. This controlled destruction is reminiscent of the autophagic elimination of certain organs that become superfluous at the onset of metamorphosis [38, 45] as well as the elimination of wing epidermal cells in the first hours of adult life [46]. A requirement for autophagy in the epidermis is consistent with previously demonstrated roles of autophagy in cellular remodeling [17, 18]. This proposed role is not inconsistent with a potential protective role of autophagy in the epidermis since a residual epidermis is maintained even in old flies.

Finally, our results imply that in the particular case of autophagy epidermal aging can be uncoupled from lifespan. The short-lived *Atg7^d77^* mutant [37] maintains a young skin. Conversely, the Progeria-like *lam^G262^* mutant, which has a short lifespan and higher epidermal autophagy, rapidly develops a very old skin. Interestingly, Progeria mice also exhibit an elevated autophagy level in the heart and other tissues [47]. In both flies and mice it remains unclear if the elevated autophagy levels are a cytoprotective reaction to Progeria or if autophagy is part of the pathology driving organ deterioration.

### Parallels between *Drosophila* and vertebrate epidermal aging

In contrast to the epidermal monolayer in flies, vertebrate skin consists of a multilayered epidermis replenished by basal stem cells and separated from an underlying dermis. The vertebrate epidermis is continuously needed to maintain a functional permeability barrier, and thus is likely to have a higher contribution to overall lifespan than the epidermis of the fly. Given these differences, how then do the changes observed in the aging *Drosophila* adult epidermis relate to vertebrate skin? A decreased integrin expression in the basal epidermis has been reported for aging human skin [13] and epidermal thinning is one of the hallmarks of aged human skin [12]. Relative to average lifespan, epidermal morphology changes in the fly have an even earlier onset than age-related changes in human skin. This difference may be explained by the fact that fly skin cells are not replenished by stem cell-mediated replacement.

Despite the morphological differences between fly and vertebrate skin, are there parallels on the molecular level that control the skin aging program? Intriguingly, mice bearing an insulin receptor substrate null mutation exhibit delayed skin aging [48] and progeroid mice with a mutant lamin exhibit hallmarks of accelerated skin aging [49]. Whether autophagy correlates with and plays a causal role in the cellular or molecular control of vertebrate skin aging *in vivo* remains to be tested. However, senescent keratinocytes in cell culture undergo autophagic cell death [21], suggesting that autophagy may play a conserved role in controlling the morphological progression of vertebrate skin aging.

A major need in the aging field is the identification of readouts of “healthspan”, which has been defined as the length of time that an individual maintains good health [6]. *Drosophila* has proven itself an important model for the identification of genes that regulate lifespan [50]. However, by contrast *Drosophila* tissue aging has only been examined in a few organ systems and behaviours including the heart, gut, muscles, nervous system, and sleep patterns [8-10]. The changes in the epidermis observed here arise earlier, can be detected using a simple visual assay, and represent a chronometer for the healthspan of an individual organism. Our model thus complements more commonly used population-based longevity assays. In addition, the epidermal aging model provides a simple platform for testing the impact of extrinsic factors such as UV radiation or caloric restriction. We expect that further molecular dissection of the epidermal aging program will reveal other regulators of tissue healthspan and how these genes are connected to organismal lifespan.

## METHODS

### Fly stocks and husbandry

Aging *Drosophila* were reared at 25 °C on standard cornmeal medium under a 12 h light-dark cycle. For consistency, only virgin females were analyzed. Aging flies were switched to new food every two to three days to avoid starvation effects.

Except where noted, *w^1118^* was used as a control strain for the rate of epidermal aging. In some cases, a Fasciclin III (FasIII)-GFP fusion transgene (YD0853; [51]) was used to visualize epidermal cell membranes. Epidermal morphology was also analyzed in long-lived *chico^1^* mutants [27], short-lived *lam^G262^* mutants [35], and *Atg7^d77^* mutants, which have reduced autophagic activity [37]. The GAL4/UAS system [52] was used for tissue-specific expression of transgenes under UAS control. The *NP2108-GAL4* driver (strain 112783; DGRC Japan) expresses in the larval and adult epidermis, as well as the persistent larval fat body. *UAS-Atg7-IR* (*CG5489-IR* #45558 from VDRC) was used for knockdown of *Atg7*. *UAS-DsRed2-Nuc* and *UAS-LC3-GFP* allowed labeling of epidermal nuclei and autophagosomes, respectively. *chico^1^/CyO*;*NP2108 -GAL4/TM6B* and *chico^1^/CyO*;*UAS-LC3-GFP* were crossed to each other to visualize autophagosomes in the *chico^1^* mutant background. Similarly, *w;lam^G262^/ CyO*;*NP2108-GAL4/TM6B* crossed to *w;lam^G262^/CyO*;*UAS-LC3-GFP* and *w;Atg7^d77^/CyO*;*NP2108-GAL4/ TM6B* crossed to *w;Atg7^d77^/CyO*;*UAS-LC3-GFP* were used to examine epidermal autophagosomes in the *lam^G262^* and *Atg7^d77^* mutant backgrounds, respectively.

### Dissection of the adult abdominal epidermis

Flies were anaesthetized and the head and appendices removed. The thorax and abdomen were pinned dorsal side up on a Sylgard (Dow Corning, Midland, Michigan, United States) dissection plate with 0.1 mm diameter dissection needles (Fine Science Tools). We covered the specimen with 1x phosphate-buffered saline (PBS), flushed out attached air bubbles, and used dissecting scissors (Fine Science Tools #15000-02, Foster City, California, United States) to make an incision along the abdominal dorsal midline. The two dorsal halves were pinned to the sides, the thorax and inner organs removed to expose the ventral abdominal epidermis. Finally, the epidermis was flattened by careful repositioning of the dissection needles. Samples were fixed for 1 to 3 h in 3.7% formaldehyde in PBS. After washing with PBS, the needles were detached and the samples transferred to 1.5 ml microtubes for subsequent staining. A more detailed protocol is available on request.

### Analysis of NP2108-GAL4 driver expression

*^1118^/*+; *NP2108-GAL4* or *UAS-2x eYFP/+; NP2108-GAL4/+* animals were collected immediately after eclosion and aged for 1, 7, or 14 days. Fat bodies were dissected and fixed in 3.7% formaldehyde in PBS for 30 minutes at room temperature. Fat bodies were then washed in PBS, mounted in Vectashield, and imaged with a Leica MZ16FA microscope with a Planapo 1.6x objective.

### Immunofluorescence

Immunostaining of adult epidermal whole-mounts was performed as described earlier for larval samples [53]. Primary antibodies were mouse anti-Fasciclin III [54] diluted 1:50, mouse anti-Coracle monoclonal antibody 9C [55] diluted 1:500 and rabbit anti-GFP antibody (A11122, Invitrogen) diluted 1:500. Secondary antibodies were Fluorescein (FITC)-conjugated goat anti-mouse (1: 250), Cy3-conjugated goat anti-mouse (1:1000) and FITC-labeled goat anti-rabbit antibody (1:250), all obtained from Jackson ImmunoResearch Laboratories Inc. All primary and secondary antibodies were diluted in PHT buffer (phosphate-buffered saline containing heat-inactivated normal goat serum and 3% Triton X100). Immuno-labeled epidermal whole mounts were imaged using a Leica MZ16FA microscope and a Planapo 1.6x objective. Photos were taken with a color Leica DFC350FX digital camera.

### Quantification of epidermal morphology

To quantify epidermal deterioration pictures of epidermal samples of different genotypes and time points, we defined four classes I to IV reflecting distinct stages of epidermal membrane labeling loss. Class I was characterized as a sample with 0 to 5% epidermal deterioration in respect to the total epidermal area, class II corresponded to 5-20%, class III to 20-50% and class IV to over 50% epidermal deterioration. Four independent individuals were asked to assign ten individual pictures for each genotype-time point combination into these pre-defined classes. The individual groupings of the four judges were then averaged for each genotype-time point combination. Subsequently the classification numbers for different genotypes and time points were compared using an exact one-sided analysis contingency table (Chi square test). Nuclear numbers and LC3-GFP signals were compared using a single-factor Anova test in Microsoft Excel.

### Autophagosome quantification

LC3-GFP-labeled epi-dermal whole mounts were visualized using an Olympus Fluoview 500 confocal microscope. Green channel settings were kept constant to allow for quantitative comparison. For each sample a Z series of optical sections in 0.7 μm increments spanning the entire depth of the epidermis and covering an area of 210 by 210 μm in the XY plane was collected and merged into a Z-stack. The resulting images were further analyzed as follows using ImagePro Analyzer 6.2. LC3-GFP signals, apparent as small punctae, were first converted to grayscale and then counted. To exclude background signals from cuticular autofluorescence, only bright punctae were counted (those >30 pixels on a 0-255 range of selected areas). Since the *lam^G262^* allele contains a GFP insertion that labels epidermal nuclei, in *lam^G262^* samples GFP-labeled nuclei were substracted from overall GFP counts in one of two ways: 1. By setting a size threshold that excluded large (area threshold here) labeled objects. 2. By then manually subtracting the remaining labeled objects that by morphological criteria and comparison to the original image seemed to result from nuclear labeling. Because some autophagosomes appear to be tightly associated with nuclei, the autophagosome counts for *lam^G262^* samples likely represent an undercount of the actual autophagosome numbers. The autophagosome numbers from all individual pictures were averaged for each genotype and time point and compared using the Student's t-test in Microsoft Excel.

### Transmission Electron Microscopy (TEM)

For transmission electron microscopy whole fly abdomens were individually dissected in 0.2 M sodium phosphate buffer (pH 7.2) and then immediately fixed for 4 h at room temperature in 3% glutaraldehyde, 2% paraformaldehyde and 2.5% dimethylsulfoxide in this same buffer. Samples were post-fixed in 1% Osmium tetroxide (Electron Microscopy Services, EMS, Hatfield, PA), briefly rinsed with water, stained with 2% aqueous uranyl acetate (EMS), dehydrated in graded ethanols, and embedded in Poly-Bed 812 resin (Polysciences Inc., Warrington, PA). Ultrathin sections were cut on a Leica EM UC6RT ultramicrotome (Leica Microsystems Inc., Bannockburn, IL), stained with lead citrate, and viewed in a JEM 1010 transmission electron microscope (JEOL, USA, Inc., Peabody, MA) at an accelerating voltage of 80 kV and 10,000x magnification. Digital images were obtained using the AMT Imaging System (Advanced Microscopy Techniques Corp, Danvers, MA). TEM pictures shown in Figure 2 and Figure S1, S3 and S4 consist of several overlapping pictures merged in Adobe Photoshop CS4. The cuticle-epidermis boundaries could be clearly distinguished by color, while the muscle-epidermis boundaries were defined by the intervening basal laminae.

## SUPPLEMENTARY FIGURES


