# Supplementary material for: Autophagy drives epidermal deterioration in a Drosophila model of tissue aging
Source: Aging (Albany NY). 2013 Apr 10;5(4):276–87. doi: 10.18632/aging.100549 (PMC3651520; doi:10.18632/aging.100549)
Supplement: Supplementary file 1 [file aging-05-276-s001.pdf]

54. Patel NH, Snow PM and Goodman CS. Characterization and cloning of fasciclin III: a glycoprotein expressed on a subset of neurons and axon pathways in *Drosophila*. Cell. 1987; 48:975-988.
55. Fehon RG, Dawson IA and Artavanis-Tsakonas S. A Drosophila homologue of membrane-skeleton protein 4.1 is associated with septate junctions and is encoded by the coracle gene. Development. 1994; 120:545-557.
56. Demerec M. 1950. Biology of *Drosophila*. (Woodbury, NY: Cold Spring Harbor Laboratory Press).

SUPPLEMENTARY FIGURES

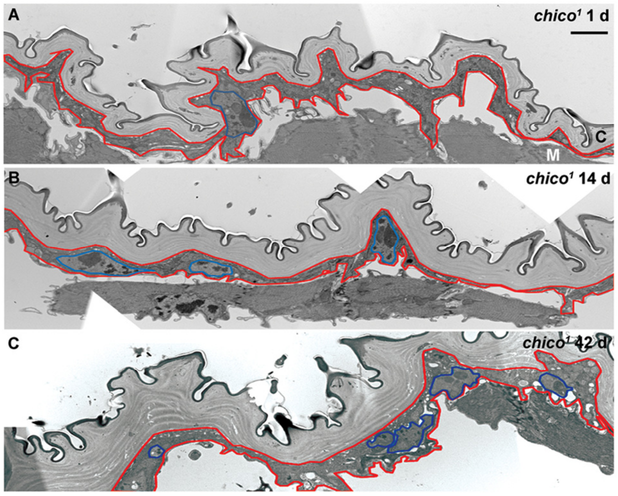

Figure S1. TEM Analysis Reveals Differential Morphology Changes in the Ventral Abdominal Epidermis of *chico*^{1} Mutants. TEM of transverse sections of ventro-lateral abdominal epidermis of: **A,** *chico*^{1} 1 d. **B,** *chico*^{1} 14 d. **C,** *chico*^{1} 42 d. Comparison of 14 d old *chico*^{1} samples (B) with age-matched controls (Figure 2C) shows that epidermal thickness is preserved. Red, epidermal boundaries. Blue, epidermal nuclei. c, cuticle; m, muscle. Bars, 2 µm.

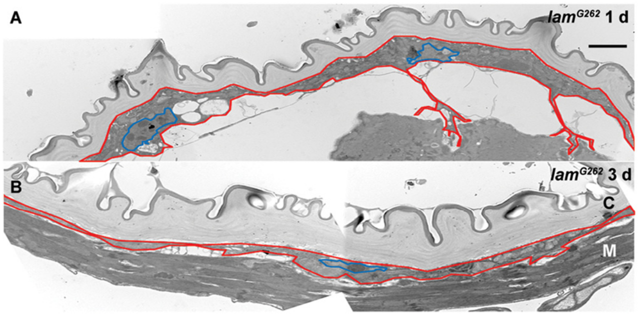

Figure S2. TEM Analysis Reveals Differential Morphology Changes in the Ventral Abdominal Epidermis of *lam*^{G262} Mutants. TEM of transverse sections of ventro-lateral abdominal epidermis of: **A,** *lam*^{G262} 1 d. **B,** *lam*^{G262} 3 d. Note the strongly condensed nuclei in 3 d old *lam*^{G262} mutants. Red, epidermal boundaries. Blue, epidermal nuclei. c, cuticle; m, muscle. Bars, 2 µm.

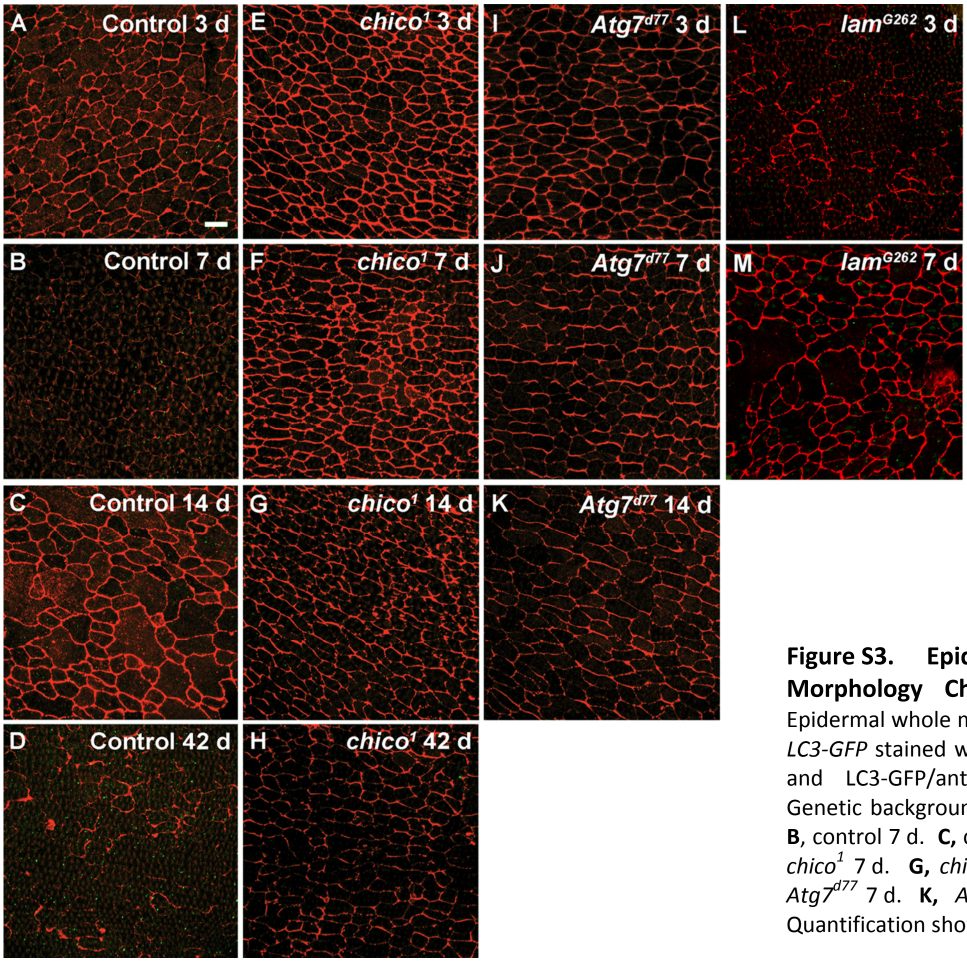

Figure S3. Epidermal Autophagy Correlates with Morphology Changes in the Aging Epidermis. A-L, Epidermal whole mounts of flies bearing *NP2108-GAL4* and *UAS-LC3-GFP* stained with anti-Fasciclin III (red) to label membranes and LC3-GFP/anti-GFP (green) to label autophagosomes. Genetic backgrounds as indicated. Bar, 20 µm. **A**, control 3 d. **B**, control 7 d. **C,** control 14 d. **D,** control 42 d. **E,** $chico^{1}$ 3 d. **F,** $chico^{1}$ 7 d. **G,** $chico^{1}$ 14 d. **H,** $chico^{1}$ 42 d. **I,** $Atg7^{d77}$ 3 d. **J,** $Atg7^{d77}$ 7 d. **K,** $Atg7^{d77}$ 14 d. **L,** lam^{G262} 3 d. M, lam^{G262} 7 d. Quantification shown in Figure 5C.

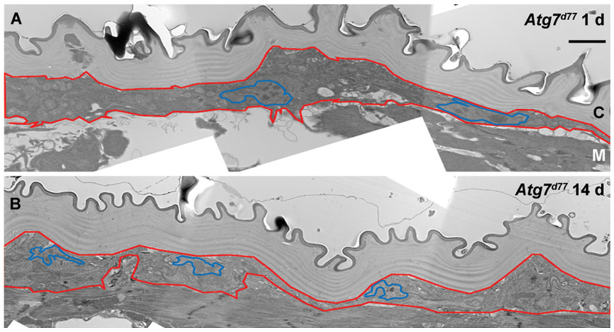

Figure S4. TEM Analysis Reveals Differential Morphology Changes in the Ventral Abdominal Epidermis of $Atg7^{d77}$ Mutants. TEM of transverse sections of ventro-lateral abdominal epidermis of: **A,** $Atg7^{d77}$ 1 d. **B,** $Atg7^{d77}$ 14 d. Comparison of 14 d old $Atg7^{d77}$ samples (B) with age-matched controls (Figure 2C) shows that epidermal thickness is preserved. Red, epidermal boundaries. Blue, epidermal nuclei. c, cuticle; m, muscle. Bars, 2 µm.

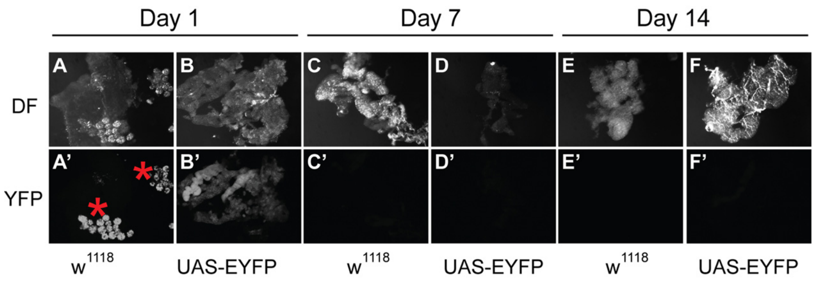

Figure S5. *NP2108-GAL4* expression does not persist in adult fat body. Fat body wholemounts from adult abdomens of the *NP2108-GAL4* driver that was crossed to either control w^{1118} animals (A, A', C, C', E, E') or *UAS-2x eYFP* (B, B', D, D', F, F'). Fat body was dissected from animals on days 1 (A,A', B, B'), 7 (C, C', D, D') or 14 (E, E', F, F') and imaged with dark field microscopy (DF; A-F) or fluorescence microscopy to detect eYFP (YFP; A'-F'). Note autofluorescence of lipids on day 1 (asterisks in A').
